# High throughput quantitative phenotyping of plant resistance using chlorophyll fluorescence image analysis

**DOI:** 10.1186/1746-4811-9-17

**Published:** 2013-06-13

**Authors:** Céline Rousseau, Etienne Belin, Edouard Bove, David Rousseau, Frédéric Fabre, Romain Berruyer, Jacky Guillaumès, Charles Manceau, Marie-Agnès Jacques, Tristan Boureau

**Affiliations:** 1 INRA, UMR1345 Institut de Recherche en Horticulture et Semences, Beaucouzé F-49071, France; 2 UMR1345 Institut de Recherche en Horticulture et Semences, SFR4207 QUASAV, PRES L’UNAM, Université d’Angers, Angers F-49045, France; 3 AgroCampus-Ouest, UMR1345 Institut de Recherche en Horticulture et Semences, Angers, F-49045, France; 4 Université d’Angers, Laboratoire d’Ingénierie des Systèmes Automatisés (LISA), Angers, F- 49000, France; 5 Present address: CREATIS; CNRS UMR5220; INSERM U630, Université de Lyon, Villeurbanne, F-69621, France; 6 INRA, UR0407 Pathologie Végétale, Montfavet, F-84140, France; 7 ANSES, Direction de la Santé des Végétaux, Angers, France; 8 Université d’ANgers, UMR1345 Institut de Recherche en Horticulture et Semences, Beaucouzé, F-49071, France

## Abstract

**Background:**

In order to select for quantitative plant resistance to pathogens, high throughput approaches that can precisely quantify disease severity are needed. Automation and use of calibrated image analysis should provide more accurate, objective and faster analyses than visual assessments. In contrast to conventional visible imaging, chlorophyll fluorescence imaging is not sensitive to environmental light variations and provides single-channel images prone to a segmentation analysis by simple thresholding approaches. Among the various parameters used in chlorophyll fluorescence imaging, the maximum quantum yield of photosystem II photochemistry (F_v_/F_m_) is well adapted to phenotyping disease severity. F_v_/F_m_ is an indicator of plant stress that displays a robust contrast between infected and healthy tissues. In the present paper, we aimed at the segmentation of F_v_/F_m_ images to quantify disease severity.

**Results:**

Based on the F_v_/F_m_ values of each pixel of the image, a thresholding approach was developed to delimit diseased areas. A first step consisted in setting up thresholds to reproduce visual observations by trained raters of symptoms caused by *Xanthomonas fuscans* subsp. *fuscans* (*Xff*) CFBP4834-R on *Phaseolus vulgaris* cv. Flavert. In order to develop a thresholding approach valuable on any cultivars or species, a second step was based on modeling pixel-wise F_v_/F_m_-distributions as mixtures of Gaussian distributions. Such a modeling may discriminate various stages of the symptom development but over-weights artifacts that can occur on mock-inoculated samples. Therefore, we developed a thresholding approach based on the probability of misclassification of a healthy pixel. Then, a clustering step is performed on the diseased areas to discriminate between various stages of alteration of plant tissues. Notably, the use of chlorophyll fluorescence imaging could detect pre-symptomatic area. The interest of this image analysis procedure for assessing the levels of quantitative resistance is illustrated with the quantitation of disease severity on five commercial varieties of bean inoculated with *Xff* CFBP4834-R.

**Conclusions:**

In this paper, we describe an image analysis procedure for quantifying the leaf area impacted by the pathogen. In a perspective of high throughput phenotyping, the procedure was automated with the software R downloadable at http://www.r-project.org/. The R script is available at http://lisa.univ-angers.fr/PHENOTIC/telechargements.html.

## Background

Quantitative phenotyping is important in plant breeding to screen accessions for resistance to pathogens. Indeed, plant resistance to pathogens may either be qualitative or quantitative. Qualitative resistance is due to the presence of single major resistance genes that confer total resistance to pathogens carrying the cognate avirulence genes. However, these monogenic total resistances are often rapidly bypassed. In contrast, resistance conferred by multiple loci exhibit quantitative phenotype and is thought to contribute to durability [1-3]. Thus quantitative phenotyping methods are necessary to ensure a good evaluation of the disease severity and to make appropriate decisions in gauging cultivar resistance in plant breeding.

Visual assessments have often been used to quantify disease severity. They require rating scales to be finely tuned, and raters to be trained, which increases considerably the cost and the time devoted to phenotyping projects. Moreover, these methods highly depend on the subjectivity of the raters and thus often lack accuracy, reproducibility and traceability [4-7].

In contrast, automated image analysis-based phenotyping provides a powerful alternative to visual assessments. Indeed, automation eventually provides a calibrated image analysis, thereby eliminating any subjectivity of the raters and ensuring reproducibility [6]. Furthermore, automation allows high throughput phenotyping. Calibrated protocols and data storage may provide useful tools for traceability or inter-laboratory comparison of the results.

Image segmentation aims at partitioning the digital image into multiple sets of pixels to select the areas of interest. In plant pathology, imaging of the symptoms has been mainly achieved using conventional color imaging. Segmentation algorithms aimed at the automated quantification of the total amount of symptoms on leaves from conventional color images [8-12]. Some of them aim at the quantification of the chlorosis or the necrosis using the differential colors of diseased leaf tissues [10,12]. Indeed, yellow and brown coloration respectively characterize chlorotic and necrotic tissues. Such automated segmentation procedures rely on color-based thresholds to discriminate diseased from healthy tissues on leaves. However, heterogeneity during exposition may alter the contrast of visible images and interfere with any color-based thresholding. Batch segmentation of visible images using color-based thresholds may thus generate numerous artifacts if light conditions during exposure are not tightly controlled. Scanner imaging of detached leaves or adjusting the color balance using a color checker may help standardizing contrasts prior to the segmentation procedures [9]. As conventional color images are typically multichannel images (for instance RGB images are composed by red, green and blue channels), they need sophisticated image analysis methods [13]. As segmentation by simple thresholding can only be applied on single channel images, conventional color images should be transformed into grayscale images prior to the segmentation. Such a transformation may result in a loss of information. Moreover, conventional color image standards aim at reproducing human vision, and thus do not directly represent the physiology of plant leaves.

Among non-conventional imaging approaches, some approaches such as thermography or chlorophyll fluorescence depict the physiology of plant leaves through single channel images [14,15]. These single channel images may easily be segmented using automated thresholding procedures for the quantification of disease severity. Chlorophyll fluorescence analysis is a non-destructive technique that has been used for imaging plant pathogen interactions [16,17] and in particular to assess the resistance of plant to pathogen [18-23]. Indeed, symptoms result from the alteration of the tissues and many pathogens target the carbon metabolism and the photosynthetic apparatus [24-26]. Among all the chlorophyll fluorescence parameters that can be estimated, the maximum quantum yield of photosystem II (PSII) photochemistry (F_v_/F_m_ = (F_m_-F_0_)/F_m_) [27] is interesting for phenotyping disease severity as it is an indicator of plant stress [17,28]. F_v_/F_m_ is a parameter calculated from two measured fluorescence parameters, F_0_ (minimum fluorescence) and F_m_ (maximum fluorescence). F_v_/F_m_ was reported to display a robust contrast between infected and healthy tissues [17,20,28,29]. Furthermore, healthy tissues were reported to yield F_v_/F_m_ values around 0.84 for numerous plant species [30,31]. When tissues are altered by biotic or abiotic stress, F_v_/F_m_ values decrease [32-34]. In many studies, mean F_v_/F_m_ measurements were used to qualitatively discriminate between diseased and healthy leaves [23,33,35-37]. However, quantitative assessments of the total diseased area on leaves require each pixel to be classified as diseased or healthy. Thresholding based on F_v_/F_m_ values may allow the segmentation of diseased areas on the imaged leaves.

Common Bacterial Blight (CBB) of bean is caused by *Xanthomonas axonopodis* pv. *phaseoli* and *X. fuscans* subsp*. fuscans* (*Xff*). These pathogens are listed by the European and Mediterranean Plant Protection Organization [38] as quarantine pathogens as CBB is the most destructive bacterial disease of the common bean *Phaseolus vulgaris* resulting in up to 60% yield losses in favorable conditions [39]. On leaves, visible symptoms start usually four to seven days after infection with pinpoint water soaked areas that enlarge and eventually form necrotic tissues surrounded by a chlorotic halo. Attack may also result in leaflet wilting and in severe cases to defoliation [38]. Furthermore, the agents of CBB may also accomplish their whole cycle in the absence of visible symptoms [40]. As for other plant bacterial diseases, no efficient chemical treatment is allowed in the European Union, and control of the disease mainly involves the use of resistant bean cultivars.

No total monogenic resistance to the agents of CBB is known in *P. vulgaris*. However, quantitative trait loci conditioning resistance to CBB have been identified and quantitative resistances may be bred into commercial cultivars of bean [41]. Therefore quantification of the total diseased area on bean leaflets is needed to monitor the resistance level of novel bean lines to the agents of CBB during the selection process. In the present study, we developed procedures for the automated segmentation of F_v_/F_m_ images in order to quantify disease severity on plant leaflets in the pathosystem *P. vulgaris*/*Xff* CFBP4834-R. At first, we explored expert-defined thresholds after visual observations to discriminate in F_v_/F_m_ images areas corresponding to necrotic, wilted, impacted tissues, and healthy tissues. Second, we tested a segmentation approach based on modeling pixel-wise F_v_/F_m_-distributions as mixtures of Gaussian distributions, each distribution representing a different stage of the alteration of plant tissues, from strongly altered to healthy tissues. Finally, we developed a thresholding approach based on the probability of misclassification of a healthy pixel into the class of diseased pixels. Then, the segmented diseased areas can be modeled as mixtures of Gaussian distributions to discriminate various stages of alteration of plant tissues, from strongly to weakly altered tissues.

## Results

### Datasets

Two datasets were used. The first one was used to setup the segmentation procedure for the quantification of symptoms by image analysis. It features images of leaflets of *P. vulgaris* cultivar (cv.) Flavert plants inoculated with the strain *Xff* CFBP4834-R or water. In this first dataset, the imaged leaflets were not detached from the plant to monitor the development of symptoms during 11 days after inoculation (dai). The same leaflets were imaged first at 1 dai then every day between 4 and 11 dai to monitor the development of the symptoms. For each leaflet, the symptomatic area was delimited either by: i) thresholding based on expert visual observations, ii), thresholding based on modeling pixel-wise F_v_/F_m_-distributions as mixtures of Gaussian distributions or iii) thresholding based on the probability of misclassification of a healthy pixel followed by a subsequent clustering of diseased pixels to describe the various stages of alteration of plant tissues.

A second dataset featured images of leaflets of five bean cultivars (cvs. Flavert, Michelet, Pike, Caprice and Wonder) inoculated with *Xff* CFBP4834-R. The image analysis procedure previously calibrated on the first dataset was applied to images belonging to this second dataset for evaluating the resistance of these commercial bean cultivars to *Xff* CFBP4834-R. In this second dataset, leaflets were detached from inoculated plants just before imaging to ease and speed up the image acquisition.

#### Thresholding based on expert visual observations

**Figure 1:**
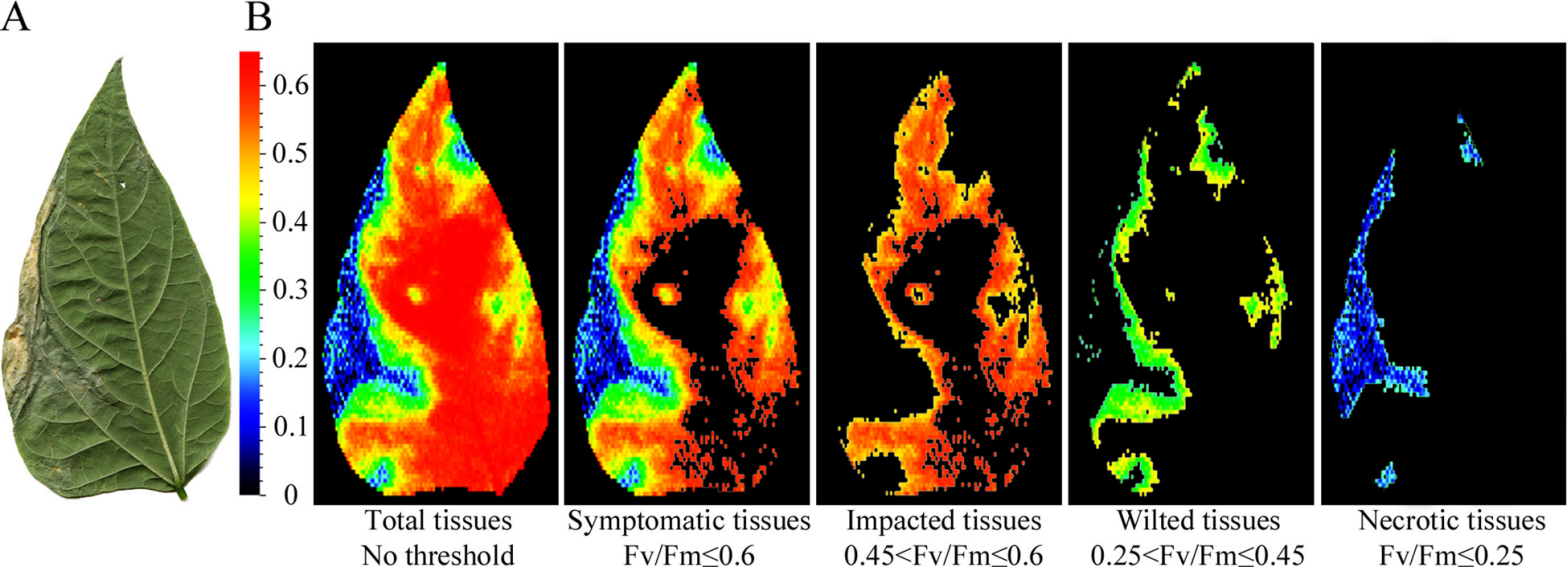
**Expert-based thresholds allow the segmentation of various stages of the symptom development. **Two weeks-old bean plants cv. Flavert were inoculated with either *Xff *CFBP4834-R (1.10^6 ^CFU ml^_1^) or mock. This leaflet inoculated with *Xff* CFBP4834-R was sampled on bean *P.vulgaris* cv. Flavert at 11 dai. **A**: visible image obtained by scanning. Necrosis is clearly visible on the left marge of the leaflet surrounded by wilted tissues. **B**: F_v_/F_m _image obtained by chlorophyll fluorescence imaging. The three stages of the symptom development, i.e. necrotic, wilted and impacted tissues, were segmented respectively with the thresholds 0.25 ≤ F_v_/F_m_, 0.25 < F_v_/F_m_ ≤ 0.45 and 0.45 < F_v_/F_m_ ≤ 0.6. Black areas represent non-selected pixels with the threshold. After the segmentation step, the proportion of pixels in each segment may be quantified.

Expert-based thresholding consisted in the comparison of conventional color images with F_v_/F_m_ images of the same leaflet by trained raters to manually define the relevant thresholds to segment the F_v_/F_m_ images. The segmented F_v_/F_m_ images should visually reproduce the distribution of symptoms as visualized on conventional color images, i.e. the various segmented parts in F_v_/F_m_ images should co-localize with the various stages of the symptom development as observed by the eye of trained raters. On bean leaflets of cv. Flavert harboring symptoms of *Xff* CFBP4834-R, we could discriminate between necrotic tissues, wilted, impacted and healthy tissues. Water soaked symptoms displayed F_v_/F_m_ values similar to that of wilted tissues, therefore both are referred to as wilted tissues. Some tissues that did not harbor any visible symptoms displayed similar F_v_/F_m_ values as chlorotic tissues, therefore both are referred to as impacted tissues. Subsequently, three F_v_/F_m_ thresholds were determined to allow the automated segmentation of necrotic, wilted, impacted and healthy tissues (Figure 1).

A training subset of images was used by the trained raters to define thresholds. The comparison of visible and F_v_/F_m_ images revealed that pixels displaying F_v_/F_m_ values inferior to 0.6 co-localized with a diseased area. F_v_/F_m_ values ranging from 0 to 0.25 co-localized with necrotic tissues, whereas F_v_/F_m_ values ranging from 0.25 to 0.45 co-localized with wilted tissues. F_v_/F_m_ values ranging from 0.45 to 0.6 corresponded to impacted tissues.

**Figure 2:**
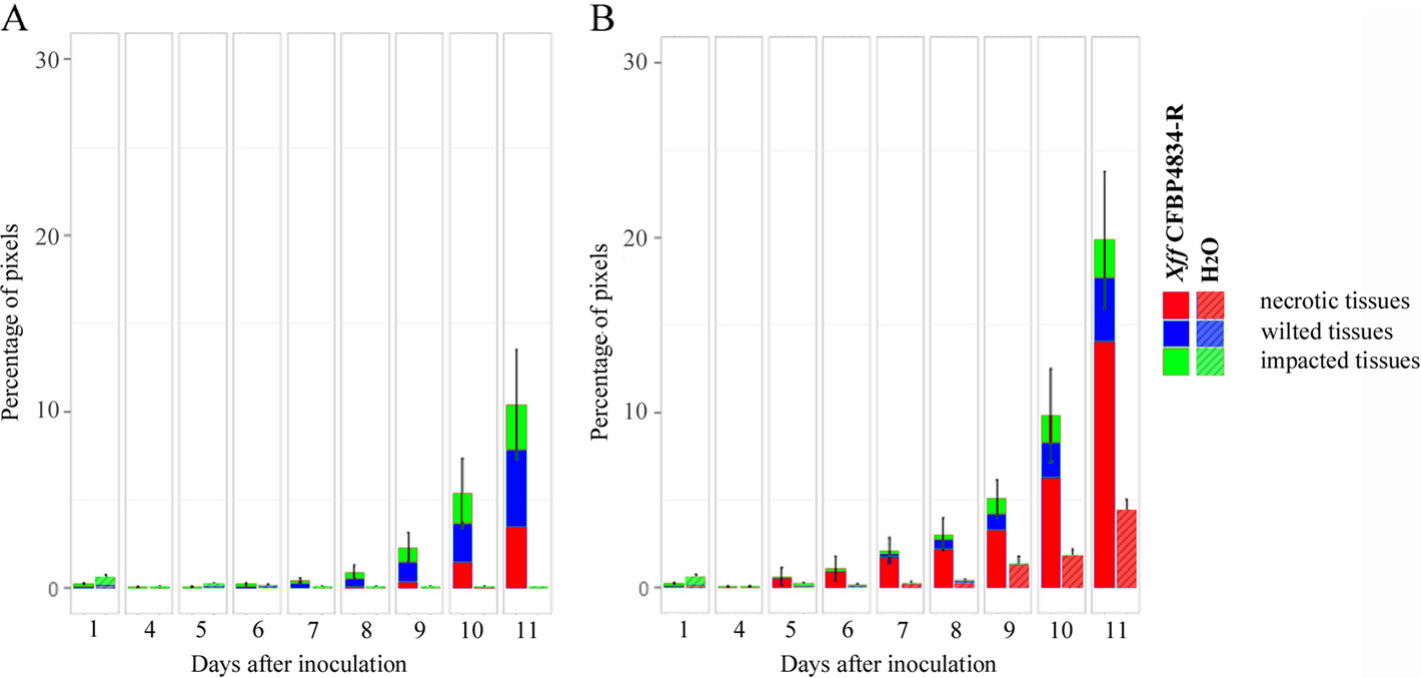
**Evolution of the proportions of necrotic, wilted and impacted tissues on bean leaflets using expert-based thresholding. **Two weeks-old bean plants cv. Flavert were inoculated with either *Xff *CFBP4834-R (1.10^6 ^CFU ml^_1^) or mock. Observations were made on bean leaflets sampled at 1 dai, and everyday after the fourth dai. Percentages of diseased tissues and standard error of the mean were calculated for 20 leaflets per sampling day. The percentages do not include (**A**) or include (**B**) the estimation of the shrinking of the leaflet. The shrinking of the leaflet is attributed to necrotic tissues.

Expert-based thresholds were applied on all F_v_/F_m_ images to quantify each stage of the symptom development. Significant symptom development began at 7 dai on leaflets inoculated with *Xff* CFBP4834-R (*p-value* < 0.01). From 9 dai on, the symptoms were predominantly composed by wilted tissues (Figure 2A).

Moreover, as in this first dataset we monitored the evolution of symptoms on the same leaflets over time, we could assess shrinking of leaflets induced by the pathogen. Shrinking corresponds to the difference between the maximum size of the leaflet and its current size at the observation timepoint. Shrinking of leaflets may represent up to 10% of the total leaflet area (Figure 2). It mostly corresponds to necrotic tissues. However, some shrinking may also originate from natural aging of leaflets as shrinking was also detected on mock-inoculated leaflets.

#### Thresholding based on modeling pixel-wise F_v_/F_m_ distributions

Abiotic stresses during the experiment may affect the physiological status of plants, which will in turn impact the F_v_/F_m_ values. In such cases, applying fixed thresholds may generate segmentation artifacts. To avoid such a drawback, mock-inoculated leaflets were used as internal daily controls to setup daily thresholds. Indeed, thresholds were defined daily to take into account the daily F_v_/F_m_ variations.

From the F_v_/F_m_ images of leaflets mock- or *Xff* CFBP4834-R-inoculated, we extracted the pixel-wise F_v_/F_m_-distributions. Analyzing these distributions may help defining appropriate non-overlapping clusters of F_v_/F_m_ values that represent various stages of alteration of plant tissues. In statistics, Gaussian mixture model (i.e. weighted sum of Gaussian distribution) is often used for clustering analysis. A clustering method proposed by Hennig [42] was tested in the present study to identify clusters of tissues according to pixel-wise F_v_/F_m_-distributions. Gaussian mixture model can be used to formalize the underlying heterogeneous distribution of F_v_/F_m_ values that are assumed to be composed of several clusters of pixels types, each cluster being modeled by a Gaussian distribution. From a practical point of view, Gaussian distributions are often too stiff to model true cluster shapes and Gaussian mixture modeling methods tend to select “larger optimal number of mixture components than what seems to be reasonable when looking to the data” [42]. Among the various methods proposed by Hennig [42] to solve this problem, we have tested the ridgeline unimodal method where clusters are merged if their fusion results in an unimodal Gaussian distribution [42,43].

In practice, using the R package MCLUST [44], the pixel-wise F_v_/F_m_-distributions were first fitted to Gaussian mixture models having from 1 to 4 components: one cluster should group pixels representing healthy tissues and three clusters should group pixels representing the various stages of the symptom development. The model best supported by the data is then determined using BIC criteria and lastly corrected by the ridgeline unimodal method [42,43]. For each pixel in the image, a probability of membership to each cluster is estimated. Clusters gathering less than 1% of the pixels were considered as not significant. Pixels initially attributed to these non-significant clusters were assigned according to their second better probability of membership.

On each image of mock-inoculated leaflets, pixel-wise F_v_/F_m_-distribution could be modeled as one single cluster per image. Predicted means of these clusters grouping pixels corresponding to healthy tissues may vary among images of mock-inoculated leaflets (e.g. from 0.71 to 0.85 at 4 dai). Hence, we calculated a confidence interval for predicted means of clusters grouping pixels corresponding to healthy tissues. Conversely, on diseased leaflets, F_v_/F_m_ values were modeled by mixtures involving more than one cluster. Among these, one cluster grouped pixels corresponding to healthy tissues, while additional clusters contained pixels corresponding to diseased areas. To discriminate between healthy and diseased tissues, a threshold based on the lower limit of the confidence interval previously defined on mock-inoculated samples was used. Subsequently, the quantification of pixels corresponding to diseased area allows the calculation of the proportion of diseased tissues on each leaflet. As expected, the proportion of diseased area on inoculated leaflets increased from 7 dai to the end of the experiment (Table 1).

**Table 1:** Quantification of the diseased tissues using the expert-, the model- and the probability-based thresholding approaches

		**1**	**4**	**5**	**6**	**7**	**8**	**9**	**10**	**11**
Expert-based thresholding	Necrotic tissues (%)	0,02	0,00	0,00	0,02	0,03	0,08	0,39	1,46,	3,44
	Wilted tissues (%)	0,06	0,01	0,01	0,06	0,19	0,49	1,02	2,15	4,41
	Impacted tissues (%)	0,16	0,05	0,06	0,13	0,20	0,37	0,90	1,74	2,56
	Total diseased tissues (%)	0,24	0,06	0,08	0,22	0,42	0,93	2,30	5,35	10,41
Model-based thresholding	Total diseased tissues (%)	0,00	5,26	5,26	23,87	42,96	5,64	35,62	49,01	30,39
Probability-based thresholding	Strong alteration (%)	0,01	0,00	0,00	0,00	0,00	0,00	0,00	1,35	2,70
	Moderate alteration (%)	0,08	0,01	0,03	0,11	0,28	0,70	1,85	2,52	6,67
	Weak alteration (%)	0,00	0,95	0,04	0,14	0,45	0,66	1,74	2,96	7,27
	Total diseased tissues (%)	0,09	0,95	0,07	0,25	0,73	1,36	3,59	6,83	16,64

Expert-based thresholding consists in defining F_v_/F_m_ thresholds that enable the selection of areas on F_v_/F_m_ images that match the various stages of the symptom development as observed by trained raters on conventional color images. Healthy, necrotic, wilted, or tissues impacted by the pathogen can be quantified.

Model-based thresholding consists in modeling the pixel-wise F_v_/F_m_-distributions extracted from each image by mixtures of Gaussian distributions. Such modeling results in the definition of clusters of pixels that correspond to various stages of the alteration of plant tissues. This step is based on the sole analysis of pixel-wise F_v_/F_m_-distributions and not on the visual observation of symptoms on conventional color images. Therefore, we use the terminology of strong, moderate and weak alteration, to emphasize that this classification is not based on visual observations, and does not necessarily correspond to the various stages of the symptom development as observed by trained raters. When applied directly without preliminary delimitation of the total diseased area, such a modeling over weights artifacts that can occur on healthy tissues, which results in a large overestimation of the proportion of diseased tissues.

Probability-based thresholds consists in the 500-quantile of the merged pixel-wise F_v_/F_m_-distributions of mock-inoculated samples. Each day of the experiment, the F_v_/F_m_ probability-based threshold allows the splitting of pixels corresponding to healthy and diseased areas. Then whithin the diseased area only, pixel-wise F_v_/F_m_-distributions are modeled as mixtures of Gaussian distributions to quantify various stages of alteration of plant tissues.

**Figure 3:**
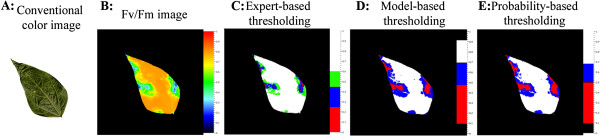
**Mapping of the diseased areas segmented using the three thresholding approaches.** Two weeks-old bean plants cv. Flavert were inoculated with either *Xff* CFBP4834-R (1.10^6 ^CFU ml^_1^) or mock. This leaflet inoculated with *Xff *CFBP4834-R was sampled on bean cv. Flavert at 11 dai. **A**: visible image obtained by scanning. **B**: F_v_/F_m_ image obtained by chlorophyll fluorescence imaging. **C-E**: Segmentation of the F_v_/F_m _image for selection of the diseased area. **C**: Expert-based thresholds are defined after comparison with visual observations by trained raters. **D**: Model-based thresholds are defined by the clustering approach on the total surface af the leaflet. **E**: Probability-based thresholds are defined on the probability that a healthy pixel is misclassified with a specificity of 0.002. The healthy tissues are represented in white. The diseased tissues are colored in red, blue or green. Defioliation spots are represented in black.

Pixels were recolored according to the cluster they belong to (Figure 3). Such a model-based clustering allows the discrimination between various stages of alteration of the plant tissues. These various stages of alteration of plant tissues strictly depend on the structure of pixel-wise F_v_/F_m_-distributions and are thus independent of any *a priori* based on visual observation of symptoms (Figure 3).

Such an approach over-weights artifacts that can occur on non-symptomatic samples. Indeed, the rule for thresholding involves the mean of the predicted distribution, but not the own F_v_/F_m_ value of each pixel. Therefore, on a mock-inoculated leaflet, if the mean of the single predicted distribution is slightly under the threshold, the whole leaflet area will be considered as diseased tissues. Such a caveat may also occur for the predicted distribution grouping pixels of healthy areas on *Xff* CFBP4834-R-inoculated leaflets, resulting in a strong overestimation of the diseased areas (Table 1).

#### Thresholding based on the probability of misclassification of a healthy pixel

To solve the caveat of the overestimation of the diseased area on inoculated leaflets, we decided to normalize on mock-inoculated samples by classifying each pixel based on the probability of misclassification of a healthy pixel. Probability-based thresholds were defined on the pixel-wise F_v_/F_m_-distributions of mock-inoculated leaflets. Thresholds were defined daily to take into account the daily F_v_/F_m_ variations. Day by day, the pixel-wise F_v_/F_m_-distributions of all the mock-inoculated leaflets were merged. The resulting distribution thus represents all the possible values for a healthy pixel, including some abnormally low F_v_/F_m_ values. The F_v_/F_m_ values corresponding to the 100-quantile, 500-quantile or 1000-quantile, i.e. the F_v_/F_m_ values splitting 1/100, 1/500 and 1/1000 of the pixels of the distribution of the mock-inoculated leaflets, were used as thresholds splitting infected and healthy tissues. For each F_v_/F_m_ image, pixels were recolored according to these thresholds. The 1/1000 threshold was too stringent, as the segmented area does not contain the totality of the visible symptom. The 1/100 threshold was not stringent enough, as randomly distributed pixels were selected in addition to symptoms. Thus, we chose the 1/500 threshold as the totality of the symptom was segmented and no randomly distributed pixels were selected (data not shown). Using such a threshold the specificity of the approach is 0.002, i.e. there was a probability of 0.002 to misclassify a healthy pixel as diseased.

**Figure 4:**
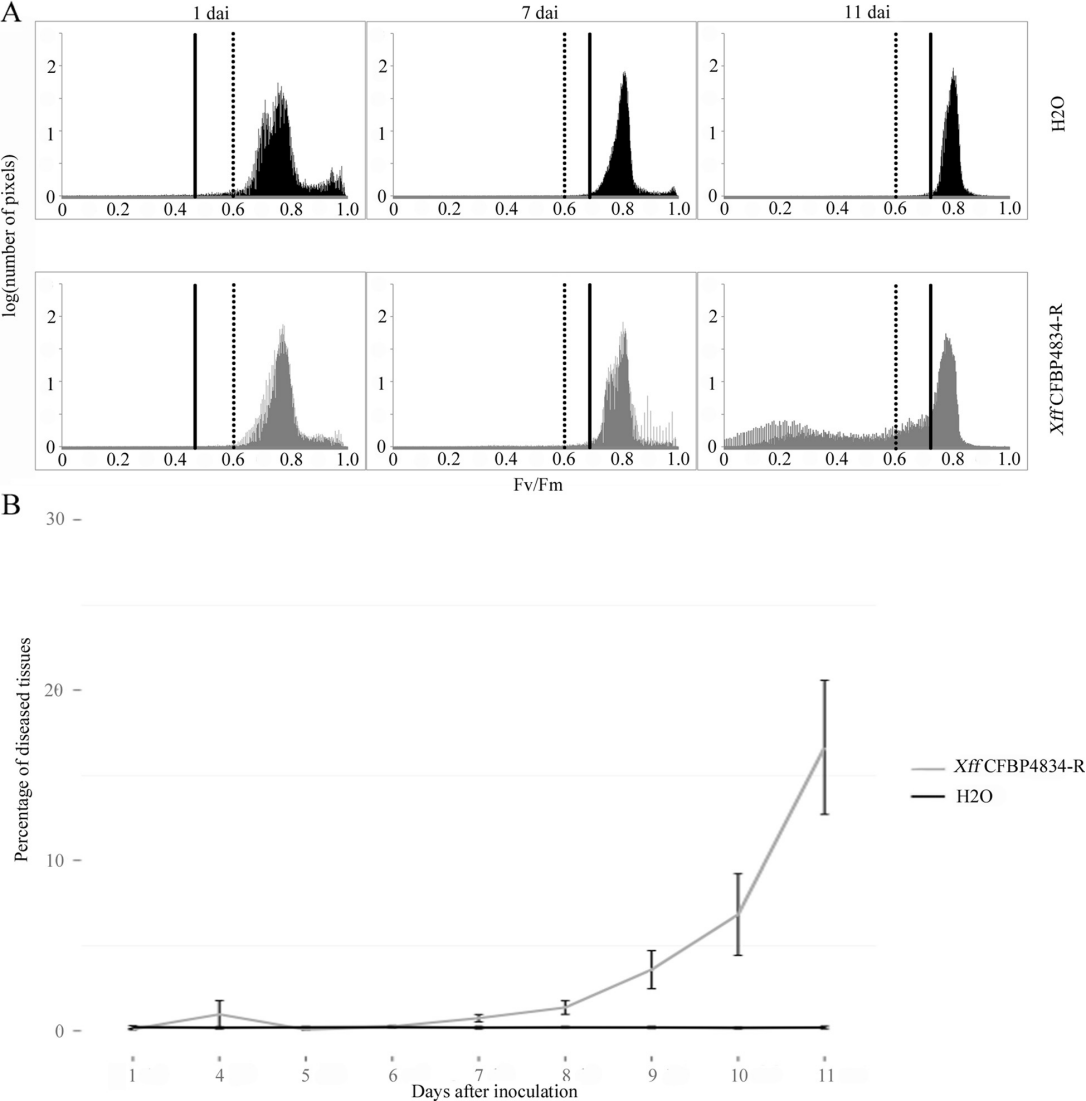
**Daily thresholds for calculation of the proportion of diseased tissues with the probability-based approach. **Two weeks-old bean plants cv. Flavert were inoculated with either *Xff *CFBP4834-R (1.10^6 ^CFU ml^_1^) or mock. **A**: Pixel-wise F_v_/F_m_-distributions at 1, 7 and 11 dai of mock-inoculated leaflets (black) and *Xff* CFBP4834-R-inoculated leaflets (grey). The expert-based thresholds (dotted bars) are fixed over the whole experiment. Conversely, the probability-based thresholds (solid bars) may vary each day of the experiment, thereby taking into account daily physiological variations of plants. **B**: Evolution of the percentage of diseased areas on mock-inoculated (black curve) and *Xff* CFBP4834-R-inoculated (grey curve) leaflets calculated with probability-based thresholding approach.

The probability-based thresholds are presented in Figure 4A by a vertical solid bar. Thresholds varied according to the day of the experiment (e.g. 0.467 at 1 dai, 0.689 at 7 dai and 0.722 at 11dai, Figure 4A) indicating that daily variations in the F_v_/F_m_ status of plants occurred during the experiment. For each leaflet, pixels exhibiting F_v_/F_m_ values lower than these probability-based thresholds were considered as diseased. Significant amounts of symptoms first arose at 7 dai (*p-value* < 0.01, Figure 4B, Table 1).

In order to discriminate between various stages of alteration of plant tissues, the pixel-wise F_v_/F_m_-distributions corresponding to the diseased tissues were extracted. Using MCLUST [44], these distributions were first fitted to Gaussian mixture models having up to 3 components to match the various stages of alteration of plant tissues (Table 1). Mapping these clusters on the recolored F_v_/F_m_ images confirmed that they match various stages of the alteration of plant tissues, i.e. weak alteration, moderate alteration, strong alteration.

#### Evaluation of the resistance of commercial bean cultivars to Xff CFBP4834-R

Five bean cultivars (cvs. Flavert, Michelet, Pike, Wonder and Caprice) were inoculated with the strain *Xff* CFBP4834-R or water. Symptoms were quantified by our image analysis procedure. The Expert-based thresholding approach was not used as the thresholds were defined from a training set of images only on cv. Flavert but not on the other cultivars. Instead, the total amount of diseased areas was determined using probability-based thresholds. Then, using a clustering approach (that does not require training datasets), the various stages of the alteration of plant tissues were discriminated and quantified.

**Figure 5:**
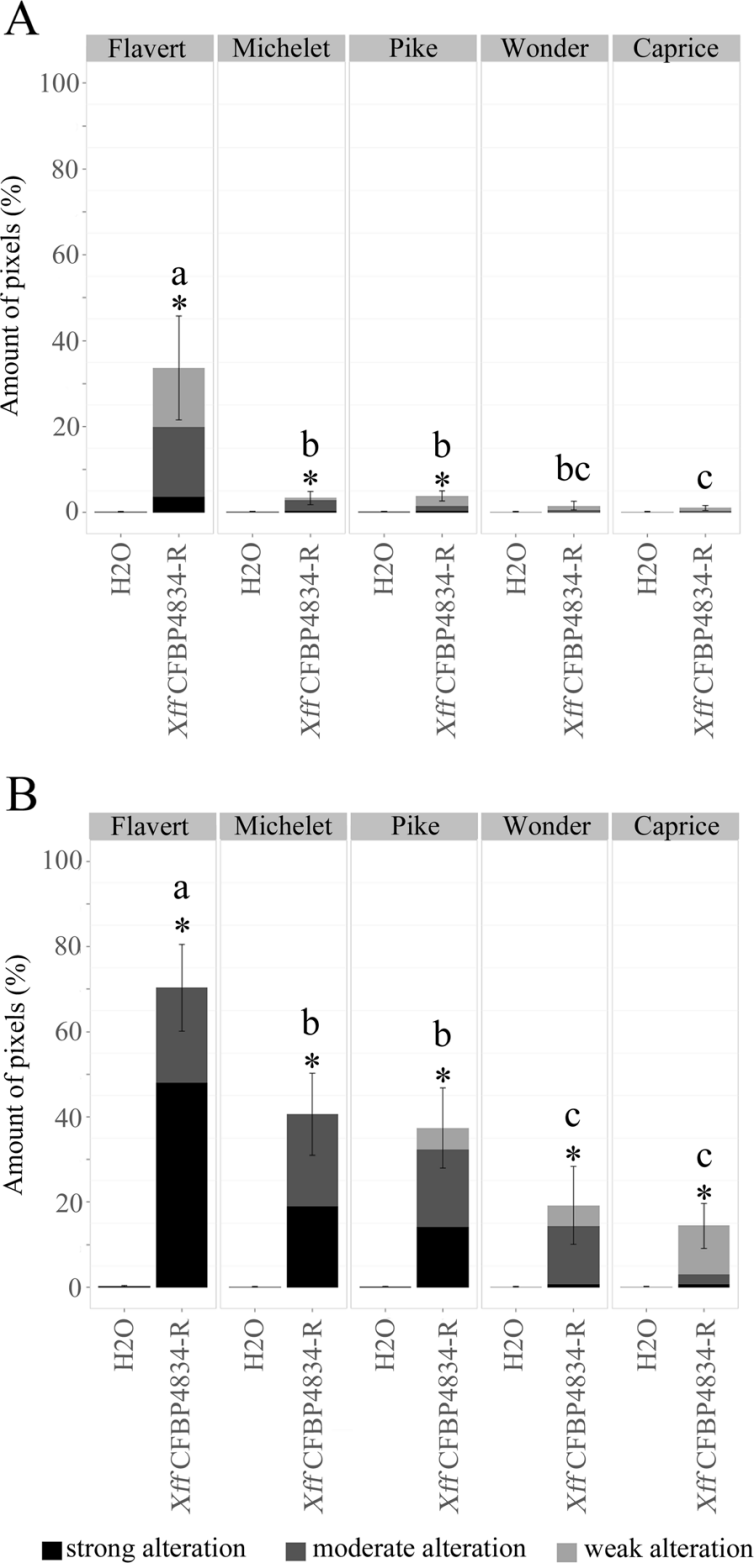
**Quantification of the proportion of diseased tissues caused by *Xff *CFBP4834-R on five cultivars of bean. **Bean leaflets of cultivars Flavert, Michelet, Pike, Wonder and Caprice were inoculated with *Xff* CFBP4834-R (1.10^6 ^CFU ml^_1^) or mock. Observations were made at 7 (**A**), and 11 dai (**B**). On each leaflet diseased area was segmented using the probability-based thresholds. Means of percentages of diseased tissues and standard error of the mean were calculated from two repeats with 4 (7 dai) and 7 (11 dai) leaflets. Treatments denoted by different letters are significantly different (*p-value* < 0.01) based on the Mann–Whitney test. Asterisks mark significant differences between the mock-inoculated and CFBP4834-R-inoculated leaflets (*p-value* < 0.01) based on the Mann–Whitney test. Clusters representing the various stages of the alteration of plant tissues were determined by a clustering approach using MCLUST [44] on the diseased area only.

F_v_/F_m_ images were taken on detached leaflets at 7 and 11 dai and the amounts of diseased tissues on leaflets were calculated (Figure 5). During the experiment, the amount of diseased tissues increased for all the cultivars tested. However, differential behaviors among the various cultivars tested could be observed. At both 7 and 11 dai, cv. Flavert exhibited a significantly higher amount of symptoms than the other bean cultivars (*p-value* < 0.05), indicating that cv. Flavert is the most sensitive cultivar to *Xff* CFBP4834-R. On the contrary, the amounts of symptoms detected on cvs. Wonder and Caprice significantly differed from the mock-inoculated samples only at 11 dai. At 11 dai, cvs. Caprice and Wonder displayed the weakest total amount of symptoms among all cultivars tested, indicating that these cultivars were the most tolerant to *Xff* CFBP4834-R in this study. At both 7 dai and 11 dai, cvs. Michelet and Pike exhibited amounts of symptoms significantly higher than mock-inoculated samples (*p-value* < 0.05). At 11 dai the total amounts of symptom detected on cvs. Michelet and Pike were significantly higher than that detected on cvs. Caprice and Wonder. Therefore, our study revealed three levels of tolerance to *Xff* CFBP4834-R at 11 dai: cv. Flavert was sensitive, cvs. Michelet and Pike were partially tolerant, and cvs. Caprice and Wonder were tolerant.

Then, the clustering approach was applied on the diseased areas to discriminate the various stages of the alteration of plant tissues i.e. weak alteration, moderate alteration and strong alteration. Therefore, an optimal number of three clusters was estimated. At 7 dai, symptoms on cv. Flavert, were essentially composed by moderately altered tissues. Some weakly altered tissues were also detected. At 11 dai, cv. Flavert exhibited a high amount of strongly altered tissues in comparison with the other cultivars. Symptoms segmented on cvs. Wonder and Caprice comprised weakly and moderately altered tissues. Cvs. Pike and Michelet presented an intermediary level of severity symptoms, i.e. moderately altered tissues and few strongly altered tissues.

## Discussion

During the last decade, non-conventional imaging techniques such as chlorophyll fluorescence imaging were used for the study of the interactions between plant and pathogens [17]. Indeed, with chlorophyll fluorescence imaging, contrasts are enhanced compared to conventional color images, and depict more accurately the physiology of plant tissues [14,15]. Chlorophyll fluorescence imaging provides images that map on leaves the variations of single parameters associated to photosynthesis. Among the various fluorescence parameters, we monitored variations in the maximum quantum yield of photosystem II photochemistry (F_v_/F_m_). We used chlorophyll fluorescence imaging to map on bean (*P. vulgaris*) leaflets the areas altered by *Xff* CFBP4834-R. It must be pointed out that most stresses that decrease leaf health will affect photosynthesis. Therefore we first checked that visible symptoms of CBB on bean leaflets co-localized with decreased values of the F_v_/F_m_ parameter. Other parameters such as F_v_/F_0_ or F_0_/F_m_ were shown to yield a high contrast between healthy tissues and tissues affected with various pathogens [20,45,46]. However, we did not treat these parameters in this study as they do not have a clear physiological significance [20].

Images based on the single F_v_/F_m_ parameter are easier to segment by thresholding approaches than conventional color images, thereby easing the image analysis process. In such a context, we developed an automated thresholding procedure to select pixels corresponding to symptoms. The respective amounts of pixels corresponding to diseased or healthy areas can then be quantified to assess the disease severity on inoculated plants. Even though the decrease in F_v_/F_m_ values due to the pathogen attack is now well documented [32,33], only few studies developed approaches for the quantification of the diseased area on leaves. Most of the studies using the F_v_/F_m_ parameter in plant pathology are based on the mean F_v_/F_m_ value over the whole image [23,33,35-37,47]. The mean F_v_/F_m_ value may qualitatively discriminate between healthy and diseased leaves, but does not quantify the amount of diseased tissues [23,33,35-37,47]. Only few studies attempted to analyze the pixel-wise F_v_/F_m_-distribution to discriminate between healthy and diseased organs using a threshold of F_v_/F_m_. For example, in the case of *Fusarium culmorum*, wheat ears were considered as infected when pixels displaying a F_v_/F_m_ value lower than 0.3 could be observed in the image [18].

In the present study, we investigated thresholding approaches for the quantification of the diseased area on infected leaves. At first, trained raters compared a subset of visible images and F_v_/F_m_ images of bean leaves of cv. Flavert inoculated with *Xff* CFBP4834-R. We could associate non-overlapping clusters of F_v_/F_m_ values to each stage of the symptom development (necrotic, wilted, impacted and healthy tissues) caused by *Xff* CFBP4834-R on *P. vulgaris* cv. Flavert. Subsequently F_v_/F_m_ thresholds could be defined to discriminate between the various stages of the symptom development. Counting the pixels associated to each F_v_/F_m_ cluster enables the quantification of the leaf area corresponding to each stage of the symptom development on cv. Flavert. Defining non-overlapping clusters of F_v_/F_m_ values to segment symptomatic areas can also be performed on a broad range of plant species to quantify areas affected by biotic or abiotic stresses. For example, on grapevine affected by lime-induced iron chlorosis, chlorotic areas displayed lower F_v_/F_m_ values than healthy tissues [48].

Interestingly, lower F_v_/F_m_ values may be observed on tissues located in the margin of symptomatic areas, but that do not display any visible symptoms. These areas evolve into symptoms over time. Therefore, as previously observed on *Arabidopsis thaliana* or *Nicotiana benthamiana* inoculated with *Pseudomonas syringae*, pre-symptomatic areas may also be phenotyped using F_v_/F_m_[33,36]. F_v_/F_m_ values in these tissues may not differ from those observed in chlorotic tissue and both chlorotic and pre-symptomatic tissues were grouped into impacted tissues in the present study. The decrease of the F_v_/F_m_ values in pre-symptomatic areas is not fully understood. Indeed, neither these areas are yet colonized by bacteria, nor can be observed increased levels of ammonia or a restricting water movement [36].

However, non-overlapping clusters matching the various stages of the symptom development should be defined by trained raters in each pathosystem studied. Indeed, F_v_/F_m_ clusters defined on Flavert do not match the visual observation on other cultivars. For example, the cluster corresponding to visually chlorotic tissues on cv. Michelet overlaps with that corresponding to necrosis on cv. Flavert (Additional file 1: Figure S1). Therefore fixed thresholds defined on a correspondence with visual observations by trained raters are valuable only within a single cultivar, and cannot be extrapolated to other cultivars. Using an expert-based thresholding approach on other cultivars needs a calibration step on a training set of pictures. Such a need is a limitation for this thresholding approach in the perspective of high throughput phenotyping, as visual assessment is time consuming.

Therefore, we aimed at defining F_v_/F_m_ thresholds that could be extrapolated to any cultivar or plant species. As a decrease of F_v_/F_m_ depicts the alteration of PSII, F_v_/F_m_ threshold can be defined independently of visual observations. Clusters of F_v_/F_m_ would depict objective stages of alteration of plant tissues. Moreover, the F_v_/F_m_ parameter may be impacted by the physiological status of plants [49,50] or abiotic stresses [16,34,51]. Fixed thresholds may therefore bias the quantification of the diseased area on leaves. Defining the thresholds on control plants for each experimental round helps avoid such a bias.

To avoid the use of fixed thresholds, we normalized our segmentation on mock-inoculated plants. We defined the threshold as the F_v_/F_m_ value under which a healthy pixel only has a probability of 0.002 to be misclassified. Such a thresholding does not allow the discrimination of various stages of the symptom development. Therefore, within the diseased area, the pixel-wise F_v_/F_m_-distribution was modeled as a mixture of predicted Gaussian distributions. Such a modeling is largely used for image analysis in medical sciences [52,53]. As well, in plant sciences such a modeling was recently applied to the automated recognition of individual Arabidopsis rosettes, in order to monitor independently the growth of each plant in the image [54]. In the present study, the clustering of pixels according to Gaussian distributions aims at describing the various stages of alteration of plant tissues without any *a priori* based on visual observations. For each F_v_/F_m_ image, a mixture of Gaussian is fitted independently, and such an approach does not need any calibration set of images. Based on these Gaussian mixture distributions, we could define non-overlapping clusters of pixels displaying similar F_v_/F_m_ values, corresponding to the various stages of alteration of plant tissues.

Pathogen attack may also result in dwarfing or shrinking of leaves. Such a phenotype is rarely quantified [9,12], but the use of non-destructive image analysis approaches may help solve such a caveat. In the present study, we monitored the size of leaflets over time. We considered the maximum size of each leaflet as a reference and the size decrease compared to this reference was considered as shrinking. Such a phenotyping is difficult to assess by visual observation only. A similar approach was used to analyze the leaf area impacted by herbivory [9]. Other approaches were proposed to evaluate the leaf deformation, for example using a sphericity index [12,55]. However, using such an index does not allow the quantification of the leaf area impacted by the shrinking.

To test the applicability of our segmentation approach for the evaluation of plant resistance, we quantified the symptoms caused by *Xff* CFBP4834-R on five commercial cultivars of bean (cvs. Flavert, Michelet, Pike, Wonder and Caprice). When looking at the total amounts of symptoms, the cv. Flavert appeared to be the most sensitive to *Xff* CFBP4834-R. The cvs. Caprice and Wonder exhibit few symptoms and can be considered as tolerant. The cvs. Michelet and Pike are impacted to an intermediary extent. On top of the amount of symptomatic tissues, selection for resistance may also focus on the stage of development of the symptom. Indeed, it may be of interest for breeders to notice that cvs. Caprice and Wonder exhibit different symptoms topologies, even though they displayed similar total amounts of symptomatic tissues.

Finally, in order to select for quantitative plant resistance to pathogens, high throughput procedures aiming at precisely quantifying disease severity need to be developed. Robotic imaging procedures can increase the number of images taken [56] but few automatic analysis of chlorophyll fluorescence images procedures are available. The procedure presented in this study was automated under R and the R script is available at http://lisa.univ-angers.fr/PHENOTIC/telechargements.html. Running our procedure on the 1080 images of our dataset, two minutes only are needed for the Expert- and Probability-based thresholding analyses. The use of MCLUST [44] to discriminate various stages of alteration of plant tissues increases up to one hour the calculation time, which remains much faster than rating disease severity by visual observations.

## Conclusions

In this paper, we described new procedures to quantify the impact of a pathogen on a plant, easy to automate, objective and accurate.

The expert-based thresholding approach aims at reproducing the visual observations. Such an approach allows the accurate quantification of the various stages of the symptoms development but needs to be calibrated by trained raters on each pathosystem. In contrast, a probability-based thresholding approach may accurately discriminate between healthy and diseased tissues. Within the diseased area, a clustering approach may accurately describe the various stages of alteration of plant tissues. This latter segmentation approach is expert-independent and is normalized on mock-inoculated plants at each day of the experiment. Moreover, the probability-based thresholding approach may allow the phenotyping of pre-symptomatic areas, which cannot be achieved by calibrating thresholds on visual observations by trained raters. A clustering approach applied on the diseased areas allows the quantification of each stage of the alteration of plant tissues. The segmentation approach developed in this study was automated using R, and the script is available at http://lisa.univ-angers.fr/PHENOTIC/telechargements.html.

Such a development of automated segmentation approach speeds up the assessment of disease severity on plants. It may reveal a significant improvement for high throughput testing of the plant resistance to pathogens during breeding.

## Methods

### Biological material

The bacterial strain *Xff* CFBP4834-R used in this study was obtained from the French Collection of Bacteria associated to Plants (CFBP, IRHS, Angers, France, http://www.angers.inra.fr/cfbp/, accession n°4885). The strain was grown at 28°C in 10% TSA medium (tryptone at 1.7 g/L, soybean peptone at 0.3 g/L, glucose at 0.25 g/L, NaCl at 0.5 g/L, K_2_HPO_4_ at 0.5 g/L, agar at 15 g/L, pH 7.2).

Beans were individually seeded in plastic pots (7 × 7 × 8 cm) containing prewetted compost (NEUHAUS HUMINSUBSTRAT *N4*, NFU 44–551). Plants were grown in a controlled climatic room at 23°C/20°C (day/night) with a photoperiod of 16 h. Plants were watered three times per week and supplemented with N-P-K (18:14:18) at 0.3 g/liter once a week.

Two sets of plants were used in this study. The first set was used to setup approaches for quantification of symptoms by image analysis and was composed by forty plants of bean Flavert. This experiment was performed three times. A second set was used to evaluate the resistance of commercial bean cultivars to *Xff* CFBP4834-R and was composed by twenty-eight plants of five cultivars of bean obtained from Vilmorin (La Ménitré, France): Flavert, Caprice, Michelet, Pike and Wonder. Twenty-eight plants of each cultivar were used. This experimentation was repeated twice.

### Pathogenicity assay

Bacterial suspensions calibrated at 1.10^8^ CFU.ml^-1^ were made by harvesting bacterial cells from agar plates and suspending them in sterile distilled water. The inoculations were made at the trifoliate step by deeping half of batch of plants during 30 seconds in the diluted bacterial suspension to 1.10^6^ CFU.ml^-1^. The other half served as control plants and was deeped in water. The first set of plants was incubated at 28°C/25°C (day/night) with a photoperiod of 16 h during 11 days and under high (70%) relative humidity. The second set of plant was incubated at 28°C/25°C (day/night) with a photoperiod of 16 h during 11 days and under high (95%) relative humidity. Plant inoculations were carried out under quarantine at UMR1345 IRHS, Centre INRA, Beaucouzé, France.

### Technical setup and image acquisition

The PSI Open FluorCam FC 800-O (PSI, Brno, Czech Republic) was used to capture chlorophyll fluorescence images and to estimate the maximum quantum yield of PSII (F_v_/F_m_) of inoculated and control leaflets. The system sensor is a CCD camera with a pixel resolution of 512 by 512 and a 12-bit dynamic. The system includes 4 LED panels divided to 2 pairs. One pair provides an orange actinic light with a wavelength of around 618 nm, with an intensity that can vary from 200 to 400 μmol/m^2^/s. It provides a 2s pulse that allows the measurement of the initial fluorescent state (F_0_). The other pair provides a saturating pulse during 1s in blue wavelength, typically 455 nm, with an intensity of up to 3000 μmol/m^2^/s. The saturating pulse allows collecting of the maximum fluorescence (F_m_). Fluorescence chlorophyll imaging was used in a dark adapted mode after a dark period of 45 min [28] to produce maps with the fluorescent quantum efficiency F_v_/F_m_ = (F_m_ - F_0_)/F_m_. For all image acquisitions, the observed leaflet is maintained horizontally for this sub-section. A kinetic analysis was performed: image acquisitions were done at 1 dai, and everyday after the fourth dai. The experiment was repeated three times, and in total 1080 images were collected. Another analysis consisted in collecting images of detached leaflets at 1, 7 and 11 dai, in total 280 images were collected. False color images representing F_v_/F_m_ values of the pixels and pixel-wise F_v_/F_m_-distributions were extracted for each leaflet. For a subset of leaflets, visible images were also taken with a digital camera to compare visual symptoms and F_v_/F_m_ values.

### Shrinking of the leaflets

The shrinking of the leaflets was calculated when a kinetic analysis was performed on each leaflet. The shrinking corresponded to the difference between the maximum size of the leaflet during the experiment and the current size of the leaflet. The shrinking of the leaflet was added to the class presenting the lower F_v_/F_m_ values. The amounts of the other stages of the symptom development were calculated using the maximum size of the leaflet.

### Thresholding based on expert visual observations

The expert-based thresholding approach consisted in the determination of values characterizing the diseased tissues. Three stages of the symptom development were discriminated: necrotic, wilted and impacted tissues. Conventional color images and F_v_/F_m_ images were manually compared by trained raters to determine clusters of F_v_/F_m_ values matching with each stage of the symptom development. On the conventional color image, the various stages of the symptom development were manually delimited and the delimitations were superimposed on the F_v_/F_m_ image. The maximum and minimum F_v_/F_m_ values of each area that co-localized with each stage of the symptom development were determined. These values corresponded to the expert-based thresholds. The amounts of pixels contained in each subgroup were calculated.

### Thresholding based on modeling pixel-wise F_v_/F_m_ distributions

The R package MCLUST [44] was run to select the number of Gaussian distributions that compose the Gaussian mixture model best supported by the data and to estimate the mean, variance and weight in the mixture distribution of each cluster. For each pixel, a probability of membership to each cluster is also returned. Gaussian mixture models including from 1 to 4 clusters with unequal variance were fitted to each leaflet pixel-wise F_v_/F_m_-distribution. The clusters representing less than 1% of the pixel-wise F_v_/F_m_-distribution were suppressed as they were considered as artifactual clusters. Pixels initially attributed to these non-significant clusters were assigned according to their second better probability of membership. The clusters of the Gaussian mixture model selected were then merged if their fusion was unimodal according to the ridgeline unimodal method implemented in the R package fpc [42,43]. A threshold was calculated for each dai to discriminate between distributions characterizing the healthy tissues and distributions characterizing the diseased tissues. The means of the clusters found on mock-inoculated samples were averaged. Then, the confidence interval was calculated. A cluster displaying a mean inferior to the threshold was considered as characterizing diseased tissues.

### Thresholding based on the probability of misclassification of a healthy pixel

The probability based-thresholds were built based a pixel-wise F_v_/F_m_-distribution resulting from the merging of the pixel-wise F_v_/F_m_-distributions of all the mock-inoculated leaflets. The thresholds corresponded to the 100-quantile, 500-quantile or 1000-quantile, i.e. the F_v_/F_m_ values splitting 1/100, 1/500 and 1/1000 of the pixels of the distribution. The thresholds were determined for each dai and each cultivar to take into consideration the possible daily variations and the differences between cultivars. A pixel displaying a F_v_/F_m_ value inferior to the threshold was considered as diseased. The total diseased proportion was then calculated for each leaflet. Then, a clustering method using the R package MCLUST [44] was performed on the diseased area detected by the Probability-based thresholds to segment the diseased area according to three stages of alteration of plant tissues. Gaussian mixture models including from 1 to 3 clusters with unequal variance were fitted to the pixel-wise F_v_/F_m_-distribution of each leaflet. In order to classify the clusters into the various stages of alteration of plant tissues, a second clustering procedure with an optimal number of clusters between 1 and 3 was performed on the means of all the clusters. The clusters were then classified according to their membership to the clusters representing the stages of alteration of plant tissues.

### Visualization of the diseased tissues and statistical test

For the three thresholding steps, the stages of the symptom development were colored using the R package EBImage [57]. The pixels were colored according to the various thresholds.

Mann–Whitney test [58] was performed to compare amounts of diseased tissues between mock-inoculated and *Xff* CFBP4834-R-inoculated leaflets and between the various cultivars of bean.

## Abbreviations

PSII: Photosystem II; Fm: Maximum fluorescence; Fv: Minimum fluorescence; Dai: Day(s) after inoculation; Xff: *Xanthomonas fuscans *subsp. *fuscans*.

## Competing interests

The authors declare that they have no competing interests.

## Authors’ contributions

CR conceived and designed the experiments, carried out acquisition of chlorophyll fluorescence data, conceived and carried out the R-analysis, interpreted the data, wrote and revised the manuscript. EBe helped setting up the acquisition of chlorophyll fluorescence data, and revised the manuscript. EBo helped setting up the acquisition of chlorophyll fluorescence data and carried out acquisition of chlorophyll fluorescence data. DR helped setting up the acquisition of chlorophyll fluorescence data, and revised the manuscript. FF designed the statistical analysis of the data and revised the manuscript. RB designed the statistical analysis of the data and revised the manuscript. JG helped in the acquisition of the chlorophyll fluorescence data. CM revised the manuscript. MAJ conceived and designed the experiments, and revised the manuscript. TB conceived and designed the experiments, carried out the acquisition of the chlorophyll fluorescence data, conceived the statistical analysis, interpreted the data, and wrote and revised the manuscript. All authors read and approved the final manuscript.

## Supplementary Material

Additional file 1: Figure S1 Expert-based thresholds needs to be calibrated on each cultivar. Symptoms of *Xff* CFBP4834-R on leaflets of cv. Flavert (A) and Michelet (B). Beans were inoculated at 1.10^6^ CFU ml^_1 ^and leaflets were sampled at 11 dai. Expert-based thresholds are defined after comparison by trained raters of F_v_/F_m _images and visual observations only on *P. vulgaris* cv. Flavert harboring symptoms of *Xff* CFBP4834-R. Using expert-based thresholds defined on cv. Flavert, chlorotic tissues on cv. Michelet are misclassified and considered as necrotic. A: visible image of a leaflet of cv. Flavert obtained by conventional color imaging and by chlorophyll fluorescence imaging. The various stages of the symptom development segmented using expert-based thresholds co-localize with visual observations. B: visible image of a leaflet of cv. Michelet obtained by conventional color imaging and by chlorophyll fluorescence imaging. The major part of the diseased tissues is composed by chlorotic tissues and misclassify as necrotic tissues with expert-based thresholds calibrated on cv. Flavert. Click here for file
