# Supplementary figures and images for: High throughput quantitative phenotyping of plant resistance using chlorophyll fluorescence image analysis
Source: Plant Methods. 2013 Jun 13;9:17. doi: 10.1186/1746-4811-9-17 (PMC3689632; doi:10.1186/1746-4811-9-17)

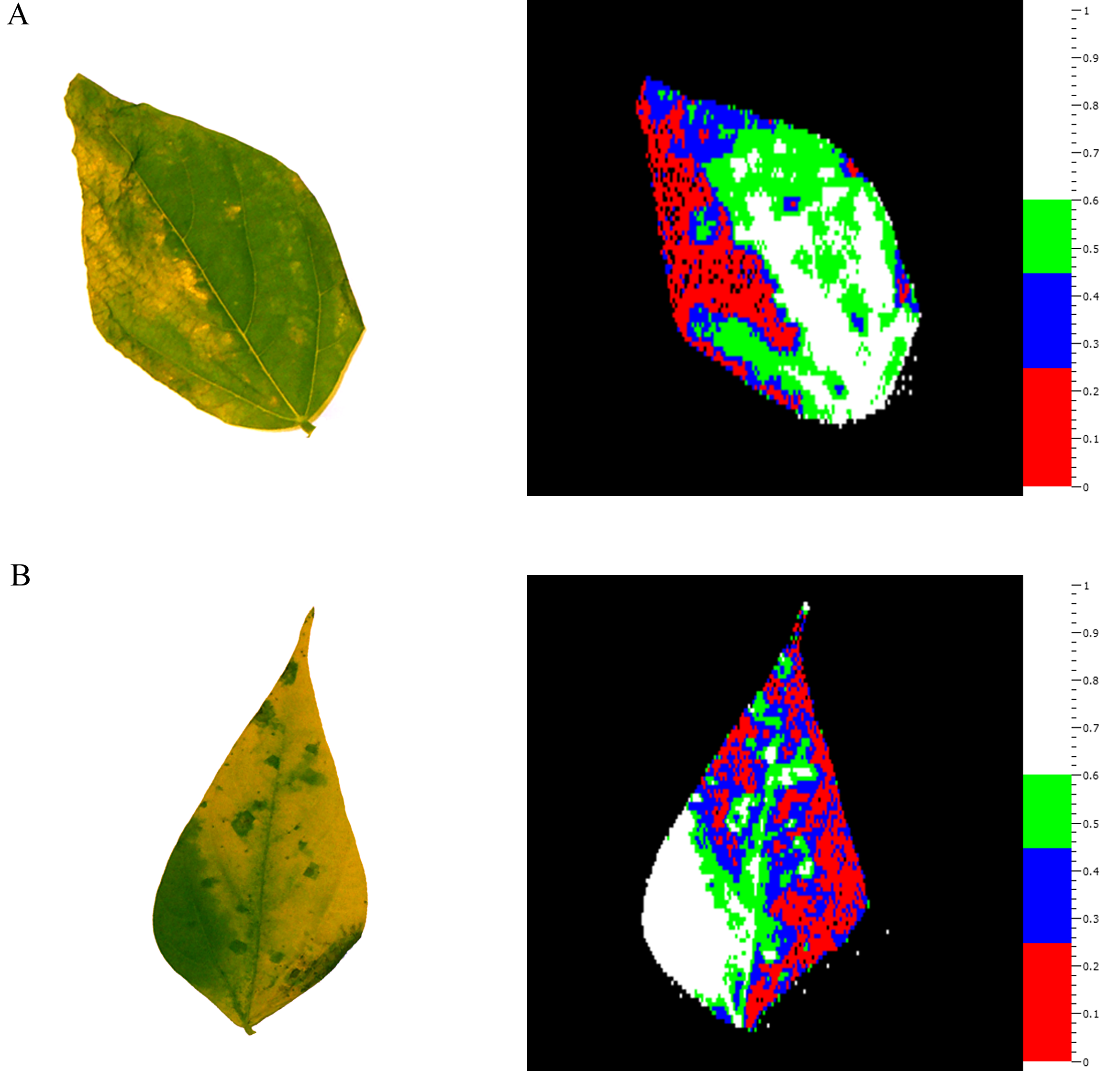

Supplement: Additional file 1: Figure S1 — Expert-based thresholds needs to be calibrated on each cultivar. Symptoms of Xff CFBP4834-R on leaflets of cv. Flavert (A) and Michelet (B). Beans were inoculated at 1.106 CFU ml_1 and leaflets were sampled at 11 dai. Expert-based thresholds are defined after comparison by trained raters of Fv/Fm images and visual observations only on P. vulgaris cv. Flavert harboring symptoms of Xff CFBP4834-R. Using expert-based thresholds defined on cv. Flavert, chlorotic tissues on cv. Michelet are misclassified and considered as necrotic. A: visible image of a leaflet of cv. Flavert obtained by conventional color imaging and by chlorophyll fluorescence imaging. The various stages of the symptom development segmented using expert-based thresholds co-localize with visual observations. B: visible image of a leaflet of cv. Michelet obtained by conventional color imaging and by chlorophyll fluorescence imaging. The major part of the diseased tissues is composed by chlorotic tissues and misclassify as necrotic tissues with expert-based thresholds calibrated on cv. Flavert. [file 1746-4811-9-17-S1.tiff]
